# Drill in Schools

**Published:** 1902-04-05

**Authors:** 


### The Hospital

A Journal of

### The Medical Sciences and Hospital Administration.

Vol. XXXII.—No. 810.] Edited by Sir Henry Burdett, K.C.B., and Solomon C. Smith, M.D., M.R.C.P. [April 5, 1902.

### Drill in Schools.

The continued stress of the war in South Africa has rendered it necessary to consider many subjects directly or indirectly connected with the national powers of offence or of defence; and among these there are few of more importance than the physical training of children—of boys, for the sake of producing healthy and intelligent men; of girls, for the sake of producing healthy and intelligent mothers. The Education Department has at last become aware of the necessity for including some provision for such training in the elementary school curriculum; and the appointment of a Royal Commission last week, to inquire into the condition of physical training in schools in Scotland, is a step of good omen in relation to the necessities of the time. An article in the "Times" of last Saturday, "from a correspondent," which appears to be the first of a series, gives an interesting description of what has already been accomplished in the district around Macclesfield by a patriotic association, and contains abundant evidence that not only His Majesty's inspectors, but also the local school managers and school teachers, are fully alive to the value of physical training, and are doing their best to promote its adoption and to improve its character. The article to which we have referred quotes a passage from a letter addressed by Lord Balfour to School Boards and school managers in Scotland, which very neatly sums up the benefits which physical training is calculated to afford. "Not only," he says, "does it tend to improve manual dexterity, and to render more alert the faculties of observation, but it is also pre-eminently useful in developing those habits of comradeship, of responsibility, and of individual resource, which are of supreme importance, not only to the nation as a whole, but to the individual pupil." It would be difficult to state the case more clearly or more concisely. Concerning the undrilled a correspondent writes to us:—

"There are few more painful spectacles than that of the average male British wage-earner on his progress towards, or on his return from, the light daily occupation, possibly that of laying 150 bricks, which it pleases him to dignify by the name of 'work.' Perhaps his chief characteristic is the total absence of anything which could be described as alertness. His slouching gait, his half-open mouth, his dangling arms, his hunched shoulders, his slow and apparently unpurposive progression, are absolutely hideous to look upon, and an intense longing to accelerate his movements by a touch with the point of some sharp instrument springs unbidden to the thoughts of every active-minded passer-by." Yet such men are the raw material of the forces which have carried the flag of England to every quarter of the globe, and who have learnt, under the agency of military drill, not only to stand at attention, but also to attend. The difference between any one of them and the smart commissionaire of the London streets appears at first sight to be so complete as almost to forbid the notion that they can even spring from the same race. And yet this difference depends upon nothing more than the habit of expanding the chest to its full proportions, and the habit of holding the muscles in a state of preparedness for action, and so braced as to be ready to respond to the mandates of the will. The dulness of the undrilled man depends largely upon the imperfect aeration of the blood which comes from imperfect or slovenly breathing; an imperfect aeration which hinders at once the promptitude of the brain to order and the promptitude of the muscles to obey. It can hardly be questioned that such a condition must greatly increase the liability to pulmonary tuberculosis, or to other forms of disease associated with depressed vitality; and it is also to a large extent responsible for the conditions which cause thousands of hobbledehoys to take sufficient interest in games to bet upon them as idle spectators, but not sufficient to engage in them with activity themselves. On the score of public health alone, therefore, the systematic drilling of school children should be encouraged, as a proceeding calculated to add to the endurance and vitality of the units of which the nation is composed, and to render them at once less liable to contract disease, and better calculated to resist it when it comes. Apart from this, the expanded chest, the well-toned muscle, and the invigorated nervous system, are imperial assets of the highest value; and are calculated to secure victory, not only in the field of battle, but also in that of industrial enterprise. Lord Balfour is probably quite right in his views of the efficacy of drill in promoting "comradeship, sense of responsibility, and individual resource," and these are the qualities which are at present most required from Englishmen in the warfare which is incidental to peace. The Empire needs men whose physical powers are fortified against disease, and whose minds are sufficiently active and recipient to feel interest in their work and curiosity with regard to the paths along which they are being guided by their political leaders. If we are to have such men, it is certain that our children must be taught to shut their mouths, to breathe through their nostrils, to fill their chests, and to keep their muscles in readiness to obey the word of command correctly and with promptitude.

